# Quantitative Analysis of Intestinal Flora of Uygur and Han Ethnic Chinese Patients with Ulcerative Colitis

**DOI:** 10.1155/2016/9186232

**Published:** 2015-12-29

**Authors:** Ping Yao, Min Cui, Haikun Wang, Hongliang Gao, Lei Wang, Tao Yang, Yongbo Cheng

**Affiliations:** Department of Gastroenterology, The First Affiliated Hospital of Xinjiang Medical University, Urumqi, Xinjiang Uygur Autonomous Region 830000, China

## Abstract

*Aim*. To study the correlation between intestinal flora and ulcerative colitis by analyzing the abundance of *Bacteroides, Fusobacterium, Clostridium, Bifidobacterium* spp., and *Faecalibacterium prausnitzii* in the intestinal of ulcerative colitis (UC) patients and healthy controls with Uygur and Han ethnic. *Methods*. Bacterial genomic DNA was extracted from fecal samples and analyzed with real-time fluorescence quantitative polymerase chain reaction (PCR) to identify the abundance of *Bacteroides, Fusobacterium, Clostridium, Bifidobacterium* spp., and *Faecalibacterium prausnitzii*. *Results*. The samples from UC patients, Uygur and Han ethnic combined, had higher abundance of *Bacteroides* (*P* = 0.026) but lower *Clostridium* (*P* = 0.004), *Bifidobacterium* spp. (*P* = 0.009), and *Faecalibacterium prausnitzii* (*P* = 0.008) than those from healthy controls. Among UC patients, *Bacteroides* population was raised in acute UC patients (*P* ≤ 0.05), while the abundance of *Clostridium*, *Bifidobacterium* spp., *Fusobacterium*, and *Faecalibacterium prausnitzii* decreased (*P* ≤ 0.05) compared with the remission. In both UC patients group and control group, no difference was observed in the abundance of these 5 bacteria between the Han and the Uygur group. *Conclusions*. Variations in the abundance of these five bacterial strains in intestines may be associated with the occurrence of UC in Uygur and Han populations; however, these variations were not associated with ethnic difference.

## 1. Introduction

In China the incidence of ulcerative colitis (UC), a type of chronic nonspecific inflammatory bowel disease, has continued to increase (11.6 per 100,000 population currently), along with advances in social development and improvements in quality of life [1–3]. To date, the causes and underlying mechanisms mediating the development and progression of UC are still not completely clear.

Many studies indicate that UC is probably related to the synergy of genetic, immunological, mental, and psychological factors, as well as the gut flora [4, 5]. Among them, the gut flora plays important roles and has become a focus of research on the digestive system [6–8]. Additionally, the incidence of UC has been shown to differ between ethnicities and regions; for example, the incidence of UC is higher in Caucasians than in blacks and in the northern United States than in the southern United States [9]. Additionally, there is a high incidence of UC in Navarre, Spain [10], but a low incidence in Uruguay [11], which may be related to the living environment, sanitary conditions, quality of life, and eating habits. Xinjiang, China, is a multiethnic region with distinctive geographical conditions, climate, and diet, resulting in unique incidences and clinical features of many diseases including UC [12], whose incidence and clinical features were reported to differ between the Uygur and Han populations, with a higher incidence and a higher percentage with moderate to severe UC diagnosis in the Uygur population. The causes of such differences are still unclear, though ethnic and regional differences may be contributing factors.

Because the gut flora is closely related to the incidence of UC [13], we hypothesized that the gut flora may differ in UC patients from different nations. The report applied 16S rRNA full-length gene technology to analyze the individual intestinal microflora and found gut microbes belonging to six phyla, including Firmicutes, Bacteroidetes, Fusobacteria, Proteobacteria, Actinobacteria, and Verrucomicrobia phyla [14]. Firmicutes and Bacteroidetes phyla have high abundance of the six phyla [15].* Bacteroides*,* Fusobacterium*, and* Clostridium* not only are phylotypes belonging to Firmicutes, Bacteroidetes, and Fusobacteria phyla, respectively, but also are the dominant genus among the three phyla. Probiotics are bacteria of human intestinal origin which are a key player in maintaining the normal function of the gastrointestinal tract [16].* Bifidobacterium* is the earliest probiotics found in human gut having the very research value. it not only is able to adjust the intestinal flora but also can improve the clinical symptoms of patients with UC [17].* Faecalibacterium prausnitzii* is the major intestinal bacterial production butyrate; improving its intestinal contents can alleviate colitis [18].

In this study, we analyzed differences in the amounts of* Bacteroides*,* Fusobacterium*,* Clostridium*,* Bifidobacterium* spp., and* Faecalibacterium prausnitzii* in the intestinal microflora of Han and Uygur ethnic patients with UC and healthy controls in order to identify correlations between intestinal flora and UC.

## 2. Methods

### 2.1. Type of Study

This case-control study was approved by the ethics committee of Xinjiang Medical University (approval number: IACUC-20121207-10).

### 2.2. Patients

Sixty patients with UC diagnosed at the Digestive System Department in the First Affiliated Hospital of Xinjiang Medical University or People's Hospital of Xinjiang were included as the case group (average age: 37.4 ± 9.6 years). Sixty age- and gender-matched individuals without digestive disease, showing normal features according to general physical examination, feces examination, and colonoscopy, were chosen as controls (average age: 39.2 ± 12.6 years). Control individuals had not taken antibiotics or probiotics for about 4 weeks. Detailed clinical information was presented in Tables 1 and 2, The gender, age, clinical stages, and scope of lesion between Uygurs and Han Chinese were balanced. Fresh fecal specimens (exposed in the air for less than 30 min) were collected, packaged into sterile tubes, and stored at −80°C for use. All subjects in both groups joined this study with informed consent.

### 2.3. Inclusion Criteria

Patients with different degrees of stomachache, diarrhea, and mucopurulent bloody stool and further diagnosed by fibrocolonoscopy and routine pathological examination (both of which met the diagnostic criteria published in the Consensus on the Standard for Diagnosis and Cure of Inflammatory Bowel Diseases in China [19]) were included in this study according to the Montreal standard to evaluate clinical performance [20].

### 2.4. Exclusion Criteria

Patients were excluded from this study if at least one of the following was true:

- Took antibiotics or probiotics within 4 weeks before specimen collection.
- Were diagnosed with infective enteritis, such as bacterial dysentery, intestinal tuberculosis, and schistosomiasis, or Crohn's disease, ischemic enteritis, radiation enteritis, irritable bowel syndrome, and colon carcinoma by fibrocolonoscopy.
- Suffered from coronary heart disease, hypertensive disease, diabetes, active pulmonary tuberculosis, or peptic ulcer.
- Took hormones, immunosuppressive agents, or sulfasalazine (SASP) during treatment.

### 2.5. Reagents and Equipment

We used a bacterial genomic DNA extraction kit (Qiagen, Germany), real-time quantitative reverse transcription polymerase chain reaction (RT-PCR) kit (Qiagen), quantitative RT-PCR instrument (SmartSpec 3000, Bio-Rad, Hercules, CA, USA), Gel Imaging System (Bio-Rad), DNA gel extraction kit (Tiangen, China), DNA molecular weight marker (Tiangen), and a refrigerated high-speed centrifuge in this study.

### 2.6. Primers

Primers for 16S rDNA of* Bifidobacterium* spp. and* Faecalibacterium prausnitzii* were designed as Rinttilä et al.'s report [21]. Primers for 16S rDNA of* Bacteroides* and* Clostridium* were designed as Liu et al.'s report [22]. Primers for 16S rDNA of* Fusobacterium* were designed as Kato et al.'s report [23]. Primer sequences are shown in Table 3. All primers were synthesized by Sangon Biotech (Shanghai, China).

### 2.7. Methods

#### 2.7.1. Bacterial DNA Extraction from Feces

Bacterial DNA was extracted from feces using a QIAamp DNA Stool Mini Kit, according to the manufacturer's instructions, and was stored at −20°C until use.

#### 2.7.2. PCR Amplification

Twenty-microliter reactions, including 10 *μ*L of 2x Taq PCR Master Mix, 0.5 *μ*L each of upstream and downstream primers, 2 *μ*L genomic DNA, and 7 *μ*L ddH_2_O, were used for amplification as follows: predenaturation at 95°C for 3 min; 35 cycles of denaturation at 95°C for 30 s, annealing at 62.8°C (*Bacteroides*), 50.8°C (*Fusobacterium*), 51.2°C (*Clostridium*), 55°C (*Bifidobacterium* spp.), or 57°C (*Faecalibacterium prausnitzii*) for 30 s, and elongation at 72°C for 1 min; and a final elongation at 72°C for 5 min. PCR products were stored at 4°C. For analysis, 10 *μ*L of each PCR product was combined with 2 *μ*L loading buffer, and the mixture was then subjected to 2.0% agarose gel electrophoresis for 30 min at a voltage of 120 V. Photographs of the gels were taken using a gel imaging system.

#### 2.7.3. Real-Time RT-PCR

Preparation of standard curves for real-time RT-PCR was performed as follows. Genomic DNA was subjected to SYBR Green I real-time quantitative RT-PCR using 20 *μ*L reaction volumes containing 10 *μ*L fluorochrome, 2 *μ*L DNA, 0.5 *μ*L each of upstream and downstream primers, and 7 *μ*L ddH_2_O. The DNA template from the control group was amplified for 16S rDNA, and PCR products were then purified and used as standards. Standard curves were created using serial dilutions (with a final concentration of 10^2^–10^7^ copies/*μ*L) and were applied as templates for amplification. A Bio-Rad IQ5 quantitative RT-PCR instrument was used for PCR amplification with the protocol same as the* PCR amplification*. The threshold cycle (Ct) was analyzed, and standard curves were automatically generated. The specificity of PCR amplification and quantification of DNA templates were analyzed on a Light Cycler PCR instrument.

Quantification of samples was carried out as follows. All samples were allocated into 4 groups: the Han-UC group (group A), the Han-control group (group B), the Uygur-UC group (group C), and the Uygur-control group (group D). Genomic DNA from feces of all groups was extracted and subjected to quantitative RT-PCR and data analysis as described above. Standards and ddH_2_O were used as control templates in each experiment, and all reactions were performed in duplicate.

### 2.8. Statistical Analysis

SPSS 19.0 was used for data analysis. Data were log transformed and presented in the form of *x*lg*x* ± *s*lg*x*. Data were subjected to tests for homogeneity of variance and normality (*P* > 0.05). Two-sample *t*-tests were used for comparisons between 2 groups, and one-way analysis of variance (ANOVA) was used for comparisons among several groups, and significant difference among means was identified by Fisher LSD test at the level of *α* = 0.05. Chi-square test was used for comparisons among count data. Differences with *P* values of less than 0.05 were considered significant.

## 3. Results

### 3.1. Analysis of Primer Specificity

**Figure 1:**
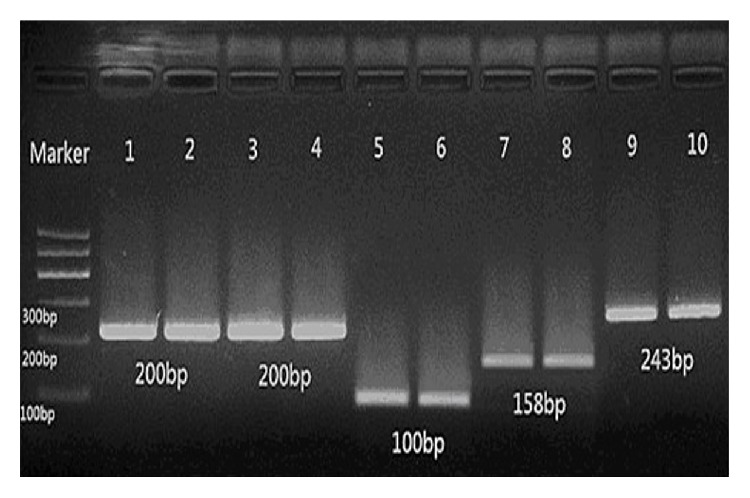
Electrophoretic gel image of five kinds of bacteria.

With 2.0% agarose gel electrophoresis, PCR products showed specific amplification with expected molecular weights, as compared to a 100 bp DNA ladder (Figure 1).

Stripes 1 and 2 show* Bacteroides* (200 bp), stripes 3 and 4 show* Clostridium* (200 bp), stripes 5 and 6 show* Fusobacterium* (100 bp), stripes 7 and 8 show* Faecalibacterium prausnitzii* (158 bp), and stripes 9 and 10 show* Bifidobacterium* spp. (243 bp).

### 3.2. Quantitative Analysis of Fecal Bacteria

Real-time quantitative RT-PCR was used to measure* Bacteroides*,* Clostridium*,* Fusobacterium*,* Faecalibacterium prausnitzii*, and* Bifidobacterium* spp. in samples from the 4 groups. The copy numbers of bacteria in each feces specimen were obtained by comparing Ct values with the corresponding standard curve.

**Figure 2:**
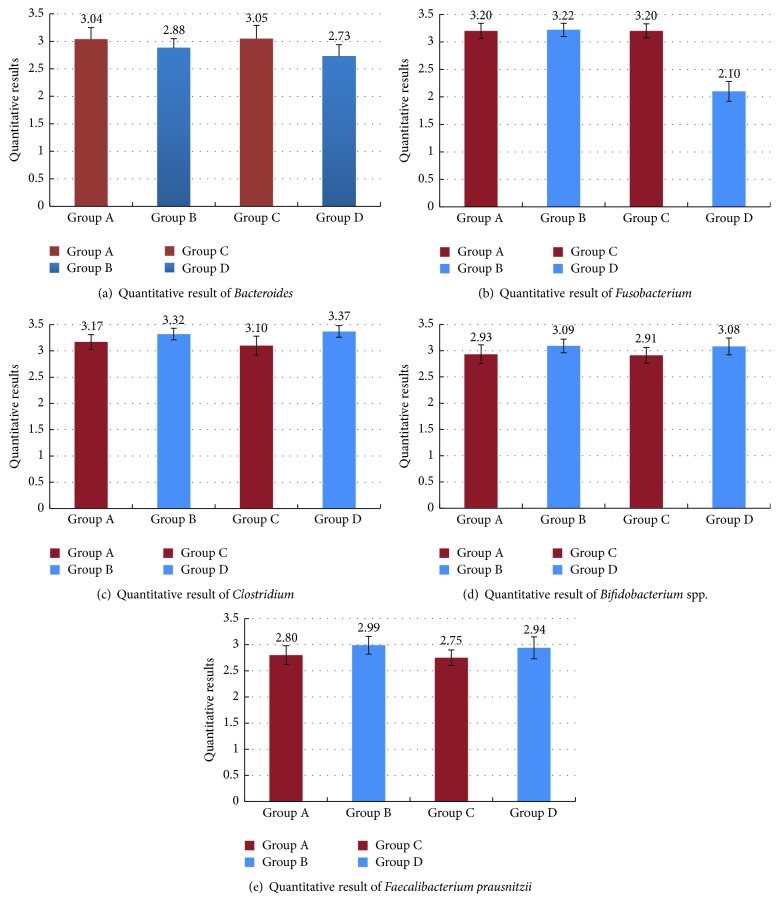
(a) Quantitative analysis of copy numbers of* Bacteroides* in the 4 groups: A, Han-UC group; B, Han-control group; C, Uygur-UC group; D, Uygur-control group. UC, ulcerative colitis. (b) Quantitative analysis of copy numbers of* Fusobacterium* in the 4 groups: A, Han-UC group; B, Han-control group; C, Uygur-UC group; D, Uygur-control group. UC, ulcerative colitis. (c) Quantitative analysis of copy numbers of* Clostridium prausnitzii* in the 4 groups: A, Han-UC group; B, Han-control group; C, Uygur-UC group; D, Uygur-control group. UC, ulcerative colitis. (d) Quantitative analysis of copy numbers of* Bifidobacterium* spp. in the 4 groups: A, Han-UC group; B, Han-control group; C, Uygur-UC group; D, Uygur-control group. UC, ulcerative colitis. (e) Quantitative analysis of copy numbers of* Faecalibacterium prausnitzii* in the 4 groups: A, Han-UC group; B, Han-control group; C, Uygur-UC group; D, Uygur-control group. UC, ulcerative colitis.

Our data indicated that there were significant differences in the copy numbers of* Bacteroides*,* Fusobacterium*,* Faecalibacterium prausnitzii*, and* Bifidobacterium* spp. between the Han-UC group and the Han-control group (*P* = 0.026, 0.004, 0.009, and 0.008, resp.), as well as between the Uygur-UC group and the Uygur-control group (*P* = 0.001, 0.001, 0.005, and 0.007, resp.). In contrast, no significant differences in* Clostridium* copy numbers were observed between the Han-UC group and the Han-control group (*P* = 0.645) or the Uygur-UC group and the Uygur-control group (*P* = 0.076). For all bacteria, no significant differences were observed between the Han-UC group and the Uygur-UC group or the Han-control group and the Uygur-control group (Figures 2(a), 2(b), 2(c), 2(d), and 2(e) and Table 4).

We also found that, in the group of UC patients, comparing with the remission,* Bacteroides* population was significantly increased in the acute UC patients (*P* ≤ 0.05), while the amounts of* Clostridium*,* Bifidobacterium* spp.,* Fusobacterium,* and* Faecalibacterium prausnitzii* significantly decreased in the acute UC patients (*P* ≤ 0.05) (Table 5).

## 4. Discussion and Conclusions

In our study, all the patients in the case group were at the initial onset stage. They were neither on any treatment nor taking any antibiotics and/or probiotics within 4 weeks before specimen collection. We quantified* Bacteroides*,* Fusobacterium*,* Clostridium*,* Bifidobacterium* spp., and* Faecalibacterium prausnitzii* 16S rDNA copy numbers in fecal specimens of Han and Uygur ethnic Chinese patients with UC and healthy controls using quantitative RT-PCR.

Contradictory to the report of previous study, no significant differences were observed between Han and Uygur populations in either the UC or control group. These data implied that changes in the quantities of these 5 bacteria were not associated with ethnic differences such as lifestyle and eating habits. Additionally, the quantity of* Bacteroides* 16S rDNA copy numbers was significantly increased in UC groups compared to that in the control group; while the quantities of* Fusobacterium*,* Faecalibacterium prausnitzii*, and* Bifidobacterium* spp. were lower in UC groups, suggesting that changes in these 4 bacteria were associated with the incidence of UC. Surprisingly, no significant differences in the copy numbers of* Clostridium* 16S rDNA were observed between any groups, and this result should be investigated further. In the group of UC patients, comparing with the remission,* Bacteroides* population was significantly increased in the acute UC patients, while the amounts of* Clostridium*,* Bifidobacterium* spp.,* Fusobacterium,* and* Faecalibacterium prausnitzii* significantly decreased in the acute UC patients, which implied that changes in the quantities of these 5 bacteria may be associated with the degree of activity of UC.

In previous studies, Verma et al. found that the number of* Lactobacilli*,* Bacteroides*,* Ruminococcus*, and* Bifidobacterium* spp. in the gut flora of Indian patients with inflammatory bowel disease decreased significantly compared to those in control patients, while the numbers of* Campylobacter*,* Methanobrevibacter*, and sulfate-reducing bacteria increased significantly compared to those in control patients [24]. Additionally, Rajilić-Stojanović et al. found that the number of* Clostridium* IV and bacteria participating in the metabolism of butyrate and propionate in the fecal flora of UC patients decreased, while the number of opportunistic bacteria, such as* Clostridium difficile*,* Campylobacter*,* Helicobacter pylori*, and* Peptostreptococcus*, increased [25]. These data indicated that an ecological imbalance occurred in the gut flora of UC patients and this imbalance might be related to the development and progression of the disease. Besides, the quantity of* Bacteroides* increased significantly in UC groups, while the quantities of* Fusobacterium*,* Faecalibacterium prausnitzii*, and* Bifidobacterium* spp. decreased significantly, partly consistent with research by Hans et al., which indicated that the changes in the numbers of these bacteria were related to UC. In a DSS mouse model, Hans et al. found that the number of* Bacteroides* increased in UC [26], with some* Bacteroides* producing enterotoxin, thereby destroying the integrity of the epithelial barrier in the intestinal mucosa, and leading to intestinal inflammation, all of which are related to UC [27] and colon cancer [28].

Studies by Kumari et al. and Kovarik et al. found that the number of butyric acid-producing bacteria decreased and the anti-inflammatory capacity of butyric acid was reduced in UC patients [29, 30]. Machiels et al. have found that the number of* Faecalibacterium prausnitzii* in UC patients decreased significantly compared to that in control individuals, and at the same time, the number of* Faecalibacterium prausnitzii* is negatively related to the degree of activity of UC [31].* Fusobacterium*,* Clostridium*, and* Faecalibacterium prausnitzii* are butyrate-producing bacteria. Butyric acid is not only the energy source of intestinal mucosa cells but also an accelerator of restoration and functional recovery of the intestinal mucosa. Additionally, butyric acid can inhibit the formation of inflammatory factors to exert its anti-inflammation functions. In a study by Macfarlane et al., flora samples in biopsy specimens from the rectums of UC patients were studied through fluorescence in situ hybridization (FISH) with a 16S rRNA probe, and the number of* Bifidobacterium* in UC patients was about 30-fold less than that in healthy individuals [32]. Zhao et al. found that* Bifidobacterium* can increase the number of CD4+CD25+Foxp3+ T cells and regulate the balance between Th1 and Th2 cells in the colonic mucosa, thereby reducing intestinal inflammation [33]. Moreover, Tanabe et al. found that* Bifidobacterium* may suppress the production of the inflammatory factor interleukin- (IL-) 17, thus attenuating intestinal inflammation [34]. Our results found that the number of* Bacteroides* increased in the UC group compared to the control group, acting in a proinflammatory manner, while the number of* Fusobacterium*,* Bifidobacterium* spp., and* Faecalibacterium prausnitzii* decreased, thus acting in an anti-inflammatory manner. Therefore, this resulting imbalance between proinflammatory and anti-inflammatory bacteria may be related to UC.

In summary, our data demonstrated that there were no statistically significant differences between Uygur and Han populations in either the UC or control group, indicating that there were no ethnic differences in bacterial loads. When disregarding ethnicity, the number of* Bacteroides* increased, while the number of* Fusobacterium*,* Bifidobacterium* spp., and* Faecalibacterium prausnitzii* decreased in the UC group compared to the control group. In UC patients, compared with the remission,* Bacteroides* population was significantly increased in the acute UC patients, while the amounts of* Clostridium*,* Bifidobacterium* spp.,* Fusobacterium,* and* Faecalibacterium prausnitzii* significantly decreased in the acute UC patients. We considered that the five kinds of bacteria have close relation to the intestinal inflammatory reaction, but the initial factors leading to a change of flora number still need further research.

Due to different methods used in separate studies and the complexity of intestinal microflora, our results differed somewhat from previous studies. However, these results provided insights into how changes in these 4 bacteria may be related to UC. There were also some limitations in this study. First, there was large variation in the types of intestinal microorganisms. Feces specimens, which contained only limited flora, were used in this study; thus, only the influence of a portion of the flora on UC was analyzed through our data analysis. Correlations between the complete intestinal flora and UC may need to be determined. Moreover, studies have reported that microorganisms in feces and in the distal bowel show 85% similarity [35]. Therefore, research on microorganisms in feces may reflect the entire intestinal flora; however, differences between microorganisms in feces and the bowel do exist [36]. Thus, analysis of microorganisms in the intestinal mucosa is necessary. Furthermore, Xinjiang is a multiethnic region, and in this study, we investigated the differences in intestinal floras of UC patients in Uygur and Han populations using only a few patients; thus, large-scale research with multiethnic individuals is required.

## What the Paper Adds to the Existing Literature on the Subject

The incidence and clinical features of ulcerative colitis (UC) in Xinjiang were reported to differ between the Uygur and Han populations [12], but the causes of such differences are still unclear. We quantitatively analyzed intestinal flora of Uygur and Han ethnic Chinese patients.

## Figures and Tables

**Table 1:** The age and gender of subjects investigated in the case and control groups.

Basic information	Uygur	Han	*χ* ^2^ or *t*	*P*
Age, years (mean ± SD)	38.33 ± 10.99	37.53 ± 10.67	0.25	0.80
Age of case group, years (mean ± SD)	38.30 ± 11.46	37.53 ± 10.69	0.27	0.79
Age of control group, years (mean ± SD)	37.77 ± 10.70	37.53 ± 10.84	0.08	0.93
Gender (male/female)	32/28	30/30	0.13	0.72
Age of case group (male/female)	16/14	15/15	0.67	0.80
Age of control group (male/female)	16/14	15/15	0.67	0.80

The “case group” means the UC patients and the “control group” means the healthy control without UC.

**Table 2:** The clinical stages and the scope of lesion in the case and control groups.

Clinical characters	Uygur	Han	*χ* ^2^	*P*
Clinical stages				
Activity	18	14	1.07	0.30
Remission	12	16	1.07	0.30
Scope of lesion				
The whole colon type	3	2	0.00	1.00
Rectal type	8	6	0.37	0.54
The left half colon type	4	8	1.67	0.20
Extensive colonic type	3	0	1.40	0.23
Rectal sigmoid type	12	14	0.27	0.60

The “case group” means the UC patients and the “control group” means the healthy control without UC.

**Table 3:** Primer sequences for 16S rDNA.

Genus	Length (bp)	Sequence (5′-3′)
*Bacteroides*	200	F: 5′-CTGAACCAGCCAAGTAGCG-3′
		R: 5′-CCGCAAACTTTCACAACTGACTTA-3′

*Fusobacterium*	100	F: 5′-CGCAGAAGGTGAAAGTCCTGTAT-3′
		R: 5′-TGGTCCTCACTGATTCACACAGA-3′

*Clostridium*	200	F: 5′-TGAAAGATGGCATCATCATTCAAC-3′
		R: 5′-GGTAACGTCATTATCTTCCCCAAA-3′

*Bifidobacterium* spp.	243	F: 5′-TCGCGTC(C/T)GGTGTGAAAG-3′
		R: 5′-CCACATCCAGC(A/G)TCCAC-3′

*Faecalibacterium prausnitzii *	158	F: 5′-CCCTTCAGTGCCGCAGT-3′
		R: 5′-GTCGCAGGATGTCAAGAC-3′

**Table 4:** The quantitative results of bacteria in feces in the case and control groups (*x*lg*x* ± *s*lg*x*).

Group	*Bacteroides*	*Fusobacterium*	*Clostridium*	*Bifidobacterium* spp.	*Faecalibacterium prausnitzii*
Han-control group	2.88 ± 0.17	3.22 ± 0.12	3.32 ± 0.11	3.09 ± 0.13	2.99 ± 0.17
Han-UC group	3.04 ± 0.21^**#**^	3.20 ± 0.14^#^	3.17 ± 0.14^#^	2.93 ± 0.18^**#**^	2.80 ± 0.18^**#**^
Uygur-control group	2.73 ± 0.21	2.10 ± 0.18	3.37 ± 0.11	3.08 ± 0.16	2.94 ± 0.21
Uygur-UC group	3.05 ± 0.24^*∗*^	3.20 ± 0.13	3.10 ± 0.18^*∗*^	2.91 ± 0.15^*∗*^	2.75 ± 0.15^*∗*^
Control group	2.81 ± 0.21	3.15 ± 0.17	3.35 ± 0.12	3.08 ± 0.14	2.95 ± 0.19
UC group	3.05 ± 0.22^▲^	3.20 ± 0.13	3.14 ± 0.17^▲^	2.92 ± 0.16^▲^	2.77 ± 0.16^▲^

^#^Compared with Han-control group, *P* ≤ 0.05; ^*∗*^compared with Uygur-control group, *P* ≤ 0.05; ^▲^compared with control group, *P* ≤ 0.05.

**Table 5:** The quantitative results of bacteria in feces in different clinical stages (*x*lg*x* ± *s*lg*x*).

Clinical stages	*Bacteroides*	*Fusobacterium*	*Clostridium*	*Bifidobacterium* spp.	*Faecalibacterium prausnitzii*
Activity	3.14 ± 0.22	3.05 ± 0.29	3.06 ± 0.22	2.77 ± 0.21	2.79 ± 0.23
Remission	2.94 ± 0.23	3.28 ± 0.20	3.20 ± 0.11	2.94 ± 0.16	2.99 ± 0.26
*t*	2.29	−2.45	−2.35	−2.39	−2.39
*P*	0.030	0.021	0.026	0.024	0.025
